# The Hypercoagulable Profile of Patients with Bone Tumors: A Pilot Observational Study Using Rotational Thromboelastometry

**DOI:** 10.3390/cancers14163930

**Published:** 2022-08-15

**Authors:** Andreas G. Tsantes, Ilectra Loukopoulou, Dimitrios V. Papadopoulos, Ioannis G. Trikoupis, Anastasios G. Roustemis, Stavros Goumenos, Rozeta Sokou, Konstantina A. Tsante, Anastasios G. Kriebardis, Panagiotis Koulouvaris, Dimitra Houhoula, Daniele Piovani, Panayiotis J. Papagelopoulos, Stefanos Bonovas, Argirios E. Tsantes

**Affiliations:** 1 Laboratory of Haematology and Blood Bank Unit, “Attiko” Hospital, School of Medicine, National and Kapodistrian University of Athens, 15772 Athens, Greece; 2 Department of Orthopaedic Surgery, Geisinger Medical Center, Danville, PA 17822, USA; 3 First Department of Orthopaedics, School of Medicine, National and Kapodistrian University of Athens, 15772 Athens, Greece; 4 Laboratory of Reliability and Quality Control in Laboratory Hematology, Department of Biomedical Science, School of Health and Caring Science, University of West Attica, 12243 Athens, Greece; 5 Department of Biomedical Sciences, Humanitas University, Pieve Emanuele, 20072 Milan, Italy; 6 IRCCS Humanitas Research Hospital, Rozzano, 20089 Milan, Italy

**Keywords:** malignancy-associated coagulopathy, rotational thromboelastometry, bone tumors, hypercoagulability, hypofibrinolysis

## Abstract

**Simple Summary:**

Malignancy-associated coagulopathy (MAC) in surgical patients with bone tumors is associated with thromboembolic complications. Rotational thromboelastometry can provide a detailed evaluation of the mechanisms involved in MAC and may allow for more effective thromboprophylactic measures. The ROTEM results indicate that the hypercoagulable state in patients with bone tumors is caused by the malignancy-associated activation of the coagulation cascade, platelet activation, and hypofibrinolysis.

**Abstract:**

Introduction: A detailed evaluation of the malignancy-associated coagulopathy (MAC) in surgical patients with bone tumors may allow for more effective thromboprophylactic measures. The purpose of this study was to assess the perioperative hemostatic changes in patients with bone tumors, using rotational thromboelastometry (ROTEM). Methods: An observational study was performed, including 50 patients with bone tumors who underwent oncologic resection and 30 healthy controls, matched for age and gender. The preoperative and postoperative laboratory evaluation of coagulation in both groups included conventional coagulation tests and a ROTEM analysis. The results of the conventional coagulation tests and the ROTEM analysis were compared between the two groups. Results: The results of the conventional coagulation tests were comparable between the tumor patients and the healthy controls. However, compared to the healthy adults, the tumor patients had lower CT (*p* < 0.001) and CFT (*p* < 0.001) values suggesting a rapid induction of the coagulation cascade, elevated A10 (*p* < 0.001) and MCF (*p* < 0.001) values indicating a higher clot strength and platelet activation, and elevated LI60 (*p* < 0.001) values indicating hypofibrinolysis in patients with bone tumors. The multiple linear regression analysis (controlling for potential confounding factors) confirmed the independent association of bone tumors with these hemostatic changes. Conclusions: Our results support the advantageous use of a ROTEM in patients with bone tumors over conventional coagulation tests because the qualitative changes in the hemostatic profile of these patients that can be detected by a ROTEM analysis cannot be identified by conventional tests. The ROTEM results indicate that the hypercoagulable state in patients with bone tumors is caused by the malignancy-associated activation of the coagulation cascade, platelet activation, and hypofibrinolysis.

## 1. Introduction

Venous thromboembolism is a potentially life-threatening condition that affects 300,000–600,000 individuals in the USA annually [1]. Both major orthopedic surgeries and neoplastic disease are associated with venous thromboembolism due to the activation of the coagulation system; therefore, patients who undergo major surgical intervention for bone tumors are at a significantly increased thrombotic risk. A detailed assessment of those hemostatic derangements that lead to hypercoagulation in these patients may allow for more effective and individualized thromboprophylactic measures following oncologic orthopedic procedures.

**Figure 1:**
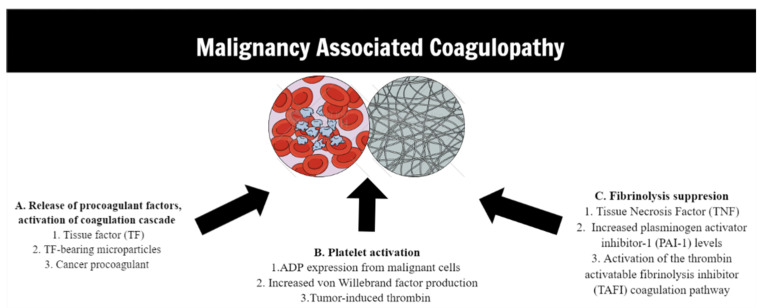
Main mechanisms involved in pathophysiology of malignancy-associated coagulopathy.

Although the association between cancer and thrombosis has been established over a century ago and is well-described by the term malignancy-associated coagulopathy (MAC), the pathogenetic mechanisms involved in this hypercoagulant state are not fully understood [2,3,4,5]. Most of these mechanisms are related to induced thrombin production due to circulating procoagulant factors, platelet activation, and fibrinolysis suppression (Figure 1). Oncologic orthopedic procedures are associated with 2–8 times higher risk for a VTE than other major orthopedic surgeries such as joint arthroplasties due to the more extensive nature of these procedures, in a preexisting hypercoagulant environment [6,7]. The overall estimated risk for a postoperative VTE following orthopedic oncologic procedures is approximately 6–16.9% [8,9,10], while certain perioperative risk factors such as the type of operation, the number of blood transfusions, and the tumor type or the presence of metastases have been also linked to a higher thrombotic risk [1].

Rotational thromboelastometry (ROTEM) and thromboelastography (TEG) are two laboratory methods that assess hemostasis by evaluating the viscoelastic properties of whole blood specimens [11]. Conventional laboratory coagulation tests such as prothrombin time (PT), the International Normalized Ratio (INR), and activated partial thromboplastin time (aPTT) are plasma-based assays that can evaluate only a specific phase of coagulation. On the other hand, viscoelastic methods such as a ROTEM have the advantage of an overall assessment of the formation and breakdown of the thrombus through a dynamic analysis of all phases of coagulation. Therefore, viscoelastic methods may be more suitable for the detection of those changes that result in hyper- or hypocoagulation in several clinical settings, such as in patients with bone tumors [12,13,14,15,16,17].

The primary aim of this study was to investigate the hemostatic changes caused by bone malignancies by comparing the ROTEM and other coagulation parameters between patients with bone tumors and healthy adults. Secondarily, we aimed to evaluate the postoperative changes in the hemostatic profile of patients with bone tumors who undergo surgery by comparing the preoperative and postoperative ROTEM parameters.

## 2. Methods

A pilot observational comparative study was conducted, including patients with bone tumors (primary and metastatic malignancies) who underwent tumor resection procedures. A matched group for age and gender was also included in the study as a control group, consisting of healthy adults who underwent minor orthopedic surgeries at the same hospital. For the matching process, each control case was selected from a group of eligible participants and was individually matched with each tumor patient for gender and age (±5 years). The study received an Institutional Review Board Approval from the hospital (ref. number: 356/9-7-2021), while informed consent was obtained from all participants. Patients and healthy adults with coagulation disorders were excluded from the study.

The prospectively collected data for each participant included demographics (age, gender, Body Mass Index (BMI), smoking, comorbidities), tumor characteristics (type of tumor, primary vs. metastatic disease, location), and laboratory parameters of coagulation. The laboratory evaluation of coagulation included conventional coagulation tests (platelet count, prothrombin time (PT), and activated partial thromboplastin time (aPTT)), and ROTEM analysis.

Laboratory testing was performed preoperatively in healthy adults to assess their baseline hemostatic profile, while in patients with bone tumors, laboratory testing was performed at 2 perioperative time points, before surgery to assess their baseline hemostatic profile and on the 2nd postoperative day to assess the postoperative hemostatic profile of these patients, under thromboprophylaxis. The patients with bone tumors were not receiving thromboprophylaxis at the time of the preoperative evaluation.

### 2.1. Sample Collection and ROTEM Analysis

A citrated tube was filled with whole blood and the sample was analyzed in a ROTEM analyzer (delta ROTEM, Tem Innovation GmbH, Munich, Germany) within 90 min from sample collection, as previously described [18,19]. It has been showed that the ROTEM results remain unaffected for blood samples stored at room temperature for up to 120 min after their collection. ROTEM analysis included evaluation of the extrinsic and intrinsic coagulation pathway through the EXTEM and INTEM assay, respectively. The following ROTEM parameters were recorded: coagulation time (CT, s), the time from the beginning of measurement to the formation of a 2 mm clot in amplitude; clot formation time (CFT, s), the time from CT to the achievement of a 20 mm clot in amplitude, clot amplitude at 10 min (A10, mm); alpha angle (a°), the angle between the horizontal centerline (*x*-axis) and the tangent to the curve of the ROTEM trace at 2 mm clot amplitude, describing the kinetics of clot formation; maximum clot firmness (MCF, mm), the maximum clot amplitude; lysis index at 60 min (LI60, %), the percentage of the residual clot firmness at 60 min in relation to MCF, reflecting the speed of fibrinolysis.

### 2.2. Statistical Analysis

Descriptive statistics of the study population for demographic data, laboratory results, and other parameters were presented as mean ± standard deviation, median, and interquartile range (IQR), or as absolute frequency and percentage, when appropriate. Variation parameters (intra-assay coefficients) for the ROTEM analysis were assessed by 15 duplicate measurements, using the same ROTEM analyzer. The 2 groups of the study, i.e., healthy adults and patients with bone tumors, were compared using the non-parametric Wilcoxon rank sum test and the Chi square test for continuous and categorical variables, respectively. In addition, the preoperative and postoperative ROTEM parameters of patients with bone tumors were compared using the non-parametric Wilcoxon signed-rank test. Last, the independent impact of neoplastic disease on the coagulation mechanism, as reflected by the ROTEM parameters, adjusted for age, gender, BMI, and smoking status, was further evaluated through multivariable linear regression analysis. The STATA software version 15.0 (Stata Corp., College Station, TX, USA) was used for data analysis. For all tests, a *p*-value less than 0.05 was considered statistically significant.

## 3. Results

**Figure 2:**
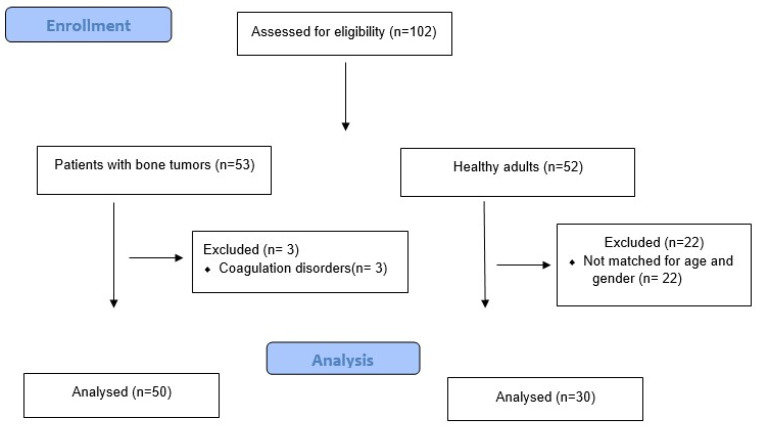
Flowchart of the study population.

Overall, 53 patients with bone tumors and 52 healthy adults were considered eligible for the study. A total of 3 patients with bone tumors were excluded because of coagulation disorders while 30 out of the 52 eligible healthy adults were matched for age and gender with each one of the tumor patients. Therefore, the final cohort consisted of 50 patients with bone tumors and 30 healthy adults (Figure 2). The median age of the patients with bone tumors and the healthy adults was 53 (IQR: 22–66) and 55 (IQR: 53–59) years, respectively (*p* = 0.62). A total of 24 (48.0%) patients were male and 26 (52.0%) were female in the tumor group, while 16 (51.6%) patients were male and 14 (48.4%) were female in the control group (*p* = 0.86). The BMI was similar for the two groups (medians: 21 in the tumor group vs. 22 in the control group; *p* = 0.10), as well as the percentage of smokers (18% in the tumor group vs. 13.3% in the control group; *p* = 0.54). Among the 50 patients with bone tumors, 38 (76.0%) had a primary bone tumor and 12 (24.0%) had metastatic bone disease. The primary bone tumors included osteosarcoma in 20 (40.0%) patients, chondrosarcoma in 13 (26.0%) patients, malignant fibrous histiocytoma in 3 (6.0%) patients, and chordoma in 2 (4.0%) patients. The primary malignancy in the patients with metastatic bone disease included breast cancer in five (10.0%) patients, lung cancer in four (8.0%) patients, and renal cancer in three (6.0%) patients. The median length of the resected bone in the patients with bone tumors was 17.0 (IQR: 15.0–18.0) cm, indicating the extensive malignant pathology in these patients with significant tumor volume. The demographic data and tumor characteristics of the study population are summarized in Table 1.

Regarding the conventional coagulation tests (Table 2), the tumor patients and the healthy adults had a comparable platelet count (medians: 260.5 × 10^3^/mL vs. 233.8 × 10^3^/mL, *p* = 0.17), PT values (medians: 12.2 s vs. 12.1 s, *p* = 0.78), and aPTT values (medians: 30.9 s vs. 31.7 s, *p* = 0.14). However, the preoperative ROTEM parameters of the tumor patients significantly differed from those of the healthy adults, indicating a hypercoagulable state in the patients with bone tumors (Table 3). Specifically, compared to the healthy adults, the tumor patients had a significantly lower EXTEM and INTEM CT (medians: 61 s vs. 65 s, *p* = 0.034, and 178 s vs. 186 s, *p* < 0.001, respectively), a lower EXTEM and INTEM CFT (medians: 51.5 s vs. 90 s, *p* < 0.001, and 64 s vs. 73 s, *p* < 0.001, respectively), a higher EXTEM and INTEM A10 (medians: 64 mm vs. 51 mm, *p* < 0.001, and 67 mm vs. 57 mm, *p* < 0.001, respectively), a higher EXTEM and INTEM MCF ( medians: 70 mm vs. 59 mm, *p* < 0.001, and 72 mm vs. 59 mm, *p* < 0.001, respectively), a higher EXTEM and INTEM alpha angle (medians: 80° vs. 70.5°, *p* < 0.001, and 82° vs. 76°, *p* < 0.001, respectively), and a higher EXTEM and INTEM LI60 (medians: 93% vs. 91%, *p* = 0.022, and 94% vs. 88%, *p* < 0.001, respectively). The variation parameters of the ROTEM analysis are summarized in Supplementary Table S1, while the duplicate measurements for the 15 specimens are presented in Supplementary Table S2.

A multivariable linear regression analysis (controlling for age, gender, BMI, and smoking status) confirmed the independent association between a hypercoagulable status and bone tumor (Table 4). Specifically, bone tumors were independently associated with a shorter EXTEM and INTEM clotting time (*p* = 0.034 and *p* = 0.013, respectively) and a shorter EXTEM and INTEM clot formation time (*p* = 0.034 and *p* = 0.013, respectively), indicating a faster initiation of the coagulation cascade due to bone malignancy. Moreover, bone tumors were independently associated with higher EXTEM and INTEM A10 (*p* < 0.001) and higher EXTEM and INTEM MCF (*p* < 0.001) values, indicating a higher clot strength in patients with bone tumors. Last, bone tumors were independently associated with higher EXTEM and INTEM LI60 values (*p* = 0.022 and *p* < 0.001, respectively), indicating lower fibrinolytic activity in patients with bone tumors.

The comparison between the preoperative and postoperative ROTEM parameters (Table 5) in the patients with bone tumors revealed that surgery resulted in an even higher coagulation potential, because the postoperative CT was lower than the preoperative CT (*p* = 0.003 for EXTEM CT and *p* = 0.013 for INTEM CT), the postoperative CFT was lower than the preoperative CFT (*p* = 0.002 for EXTEM CFT and *p* = 0.018 for INTEM CFT), the postoperative A10 was higher than the preoperative A10 (*p* = 0.007 for EXTEM A10 and *p* = 0.009 for INTEM A10), and the postoperative MCF was higher than the preoperative MCF (*p* = 0.003 for EXTEM MCF and *p* = 0.005 for INTEM MCF).

Another interesting finding of our study was that metastatic bone disease was associated with a more hypercoagulable profile based on the ROTEM parameters than the primary bone tumors, probably due to the systemic spread of malignant cells. Specifically, the patients with metastatic bone disease had a higher EXTEM and INTEM A10 (*p* < 0.001 for EXTEM A10 and *p* = 0.040 for INTEM A10) and a higher EXTEM and INTEM MCF (*p* < 0.001 for both EXTEM and INTEM MCF) compared to the patients with primary bone tumors, suggesting a higher clot strength in metastatic bone disease (Table 6).

## 4. Discussion

The risk of a clinically significant VTE is four-to-seven times higher in oncologic patients compared to the general population [20]. Many causes such as malignancy-induced platelet activation have been implicated in cancer-associated thrombosis while various factors related to surgery, such as endothelial damage and massive release of tissue factor, also add to the overall increased thrombotic risk [21,22]. Further understanding the hemostatic derangements that result in the prothrombotic state of surgical oncologic patients may allow for the development of targeted thrombophylactic measures focused on specific aspects of the hemostatic mechanism. The results of this study indicate that bone tumors are associated with accelerated clot formation and a higher clot strength, as reflected by the lower CT and CFT values and the higher A10 and MCF values in the patients with bone tumors. Moreover, the elevated LI60 values in the tumor patients compared to healthy adults indicate that bone tumors are also associated with lower fibrinolytic activity.

The rapid clot formation in MAC can be attributed to many circulating procoagulant factors that activate factor X and result in the rapid activation of the coagulation cascade. These procoagulant factors include the tissue factor (TF), a protein released by endothelial cells, monocytes, and malignant cells; the TF-bearing microparticles (cancer-associated procoagulant proteins that free float in the plasma of oncology patients and also interact with factor X); and the cancer procoagulant, a molecule that has been found only in fetal and malignant tissue. The release of these procoagulant factors and the enhanced activation of coagulation result in the rapid production of high amounts of thrombin. The acceleration of the initial phases of coagulation as reflected by the shortened clotting time and clot formation time has also been showed in other studies evaluating the hemostatic profile of oncologic patients, using viscoelastic methods. Thouk et al. evaluated the hemostatic profile of 32 patients with prostate cancer by comparing their TEG parameters with those of 8 healthy adults [23]. The authors found that the reaction time (R time), a TEG parameter similar to the ROTEM CT, was also significantly shorter in cancer patients. In another study using TEG to evaluate the hemostatic profile of patients with malignancies, Wehrum et al. also found that patients with gynecologic malignancies had a shorter reaction time and a shorter kinetics time (K time, a TEG parameter similar to the ROTEM CFT) compared to the age-matched controls, indicating rapid clot formation due to malignancy as in our study [24]. Last, Akay et al. reported that the hypercoagulability in MAC is partially caused by accelerated clot formation, as the authors found decreased CFT levels in 78 cancer patients compared to 16 age-matched healthy adults [25].

Malignant cells release activators that stimulate platelets and enhance clot strength. Increased ADP expression from malignant cells, increased von Willebrand factor production, and tumor-induced thrombin are some of the main mechanisms involved in platelet activation due to malignancy. The elevated MCF and A10 values that were seen in our group of tumor patients could be attributed to the enhanced platelet activation. Moreover, the elevated MCF and A10 levels, despite the similar platelet count in tumor patients and healthy adults, highlight the advantage of viscoelastic methods over conventional coagulation tests to identify those qualitative changes in MAC that lead to hypercoagulability and are not detectable by the conventional coagulation studies. Elevated MCF and A10 values in cancer patients have also been reported in other studies. Davies et al. compared the hemostatic profile of 67 lung cancer patients with that of 72 age-matched healthy controls, using a ROTEM analysis [26]. Similar to our findings, the authors of this study reported elevated MCF levels for both the EXTEM and INTEM assays in cancer patients compared to healthy adults. It is also noteworthy that Davies et al. reported that most patients had normal ROTEM values based on what is considered a normal reference range. This is in line with our findings, because most of the ROTEM parameters, such as the MCF, were falling within the established reference range, while only A10 was abnormal in most cancer patients. These findings indicate that the current definitions for hypercoagulability based on ROTEM parameters may be inadequate to describe certain clinical settings. In another study by Quan et al., the authors also evaluated the hemostatic profile of lung cancer patients, using TEG [27]. The authors reported that the Maximum Amplitude (MA), a TEG parameter similar to the ROTEM MCF, was significantly higher in cancer patients compared to age- and gender-matched controls and that the detection rate of hypercoagulability was higher for TEG compared to conventional coagulation tests. These findings are similar to ours where the MCF was also higher in cancer patients, indicating a higher clot strength, while conventional coagulation studies failed to identify the hypercoagulant state of cancer patients.

The suppression of fibrinolysis in MAC is mediated through several proteins that are released by malignant cells and downregulate fibrinolytic activity, such as the Tissue Necrosis Factor (TNF) [5]. Moreover, the MET oncogene that is expressed in many cancer types also results in fibrinolysis inhibition because it has been associated with increased plasminogen activator inhibitor-1 (PAI-1) levels [22]. Another mechanism of fibrinolysis suppression in cancer patients includes the dysregulation of the thrombomodulin–thrombin complex that leads to the activation of the thrombin activatable fibrinolysis inhibitor (TAFI) coagulation pathway [5]. The suppression of the fibrinolytic activity due to malignancy was evident in our study, because the patients with bone tumors had higher LI60% levels compared to the healthy adults. However, fibrinolysis shutdown was not evident in these patients, based on the ROTEM criteria for fibrinolysis shutdown [28,29,30,31]. In line with our findings, a lower fibrinolytic activity, as reflected by the TEG parameters, was also identified in a study by Moore et al. in which a significantly higher number of patients with pancreatic cancer were hypercoagulable based on the LY30 parameter (clot lysis at 30 min after maximum clot strength) compared to those without pancreatic cancer [32].

There are some studies that tried to evaluate whether the ROTEM parameters can detect the association between hypercoagulability and the development of thromboembolic complications following surgeries [13,33]. Hincker et al. investigated the prognostic ability of the ROTEM results for postoperative thromboembolic complications in 313 patients who underwent major noncardiac surgeries, including oncological surgical resections [33]. The authors of this study found that several ROTEM parameters, such as the INTEM A10, had high prognostic ability for postoperative thromboembolic complications. In another study by our group, the accuracy of the ROTEM parameters to detect the association between the hypercoagulability and thromboembolic complications after hip fracture surgeries was also evaluated [13]. The results of this study indicated that the results of the ROTEM analysis are associated with the development of symptomatic thromboembolic complications after hip fracture surgeries, with the preoperative INTEM CFT having the highest accuracy in terms of the association with thromboembolic complications.

There are some limitations of this study that must be addressed. First, the number of included patients is relatively small and no power analysis was performed to estimate the population size of the two groups. However, there is lack of relevant information on patients with bone tumors, because this is the first observational study comparing the ROTEM parameters in patients with bone tumors and healthy adults. Second, certain clinical outcomes, such as thromboembolic events, were not evaluated in this study due to the small number of patients which does not allow for any statistical comparison between patients with and without VTE events in order to draw safe conclusions regarding the association between ROTEM parameters and VTE. Third, even though this is not a randomized study and several factors, such as demographics, can confound the association between the ROTEM parameters and bone tumors, the two included groups were matched for age and gender. Moreover, a linear regression analysis was performed to adjust for other confounding factors, such as smoking status and BMI. Last, certain comorbidities can affect the coagulation status of healthy adults and patients with bone tumors, and the evaluated association between the ROTEM parameters and bone tumors was not adjusted for any comorbidities. However, in order to decrease the possibility of any bias due to different comorbidities in the two groups, we aimed to exclude patients and healthy adults with congenital and acquired coagulopathies, because these pathologies clearly affect the ROTEM parameters.

## 5. Conclusions

Conclusively, rotational thromboelastometry can provide a detailed evaluation of the hemostatic derangements that occur in patients with bone tumors. Specifically, the shorter clotting time and clot formation time indicate a rapid initiation of the coagulation cascade due to the release of procoagulant factors by malignant cells, while platelet activation results in enhanced clot strength as reflected by the elevated A10 and MCF levels. Malignancy-associated hypofibrinolysis was also evident, because patients with bone tumors had elevated LI60 levels compared to healthy controls. Moreover, conventional coagulation studies are less suitable for the perioperative monitoring of patients with bone tumors because they fail to detect the qualitative changes in the hemostatic profile of these patients that lead to a hypercoagulant state. Last, larger studies are needed in order to re-evaluate the reference range of the ROTEM parameters in cancer patients because the hypercoagulable state of these patients is associated with the ROTEM values that fall within the established normal reference range, while further research is also needed in order to investigate the association of the ROTEM parameters with the development of thromboembolic complications.

## Figures and Tables

**Table 1:** Characteristics of the study population.

	Patients with Tumors (*n* = 50)	Control Group (*n* = 30)	*p*-Value
Age (years)	47.9± 22.9, 53 (22–66)	54.8 ± 4.3, 55 (53–59)	0.62
Gender (males %)	24 (48.0)	16 (51.6)	0.86
BMI (kgr/m^2^)	20.7 ± 2.1, 21.0 (19.0–22.0)	21.8 ± 2.3, 22 (20–23)	0.10
Smoking	9 (18.0)	4 (13.3)	0.54
Tumor Primary Metastatic	38 (76.0) 12 (24.0)	-	
Location Proximal femur Distal femur Proximal tibia	15 (30.0) 32 (64.0) 3 (6.0)	-	

Data are presented as means ± SD, medians, and interquartile ranges (IQR), or as absolute values (percentages), when appropriate. The nonparametric Wilcoxon rank sum test and the Chi square test were used for the comparison between the 2 groups. Abbreviations: BMI, Body Mass Index.

**Table 2:** Conventional coagulation assays of the study cohort.

Variables	Patients with Tumors (*n* = 50)	Control Group (*n* = 30)	*p*-Value
PLTs (count × 10^3^/mL)	260.5 ± 101.3, 259.0 (182.0–324.0)	233.8 ± 20.8, 231.0 (225.0–245.0)	0.17
aPTT (s)	30.9 ± 3.8, 30.6 (28.5–30.6)	31.7 ± 2.8, 31.6 (30.0–34.0)	0.14
PT (s)	12.2 ± 1.6, 11.8 (11.2–12.8)	12.1 ± 1.8, 12.0 (10.4–13.2)	0.78

Data are presented as means ± SD, medians, and interquartile ranges (IQR). The nonparametric Wilcoxon rank sum test was used for the comparison between the 2 groups. Abbreviations: PLTs, platelets; aPTT, activated partial thromboplastin time; PT, prothrombin time.

**Table 3:** Preoperative ROTEM results of patients with musculoskeletal tumors and ROTEM results of healthy adults.

Variables	Patients with Tumors (*n* = 50)	Control Group (*n* = 30)	*p*-Value
EXTEM CT (s)	61.2 ± 6.2, 61 (58.0–65.0)	64.9 ± 7.3, 65.0 (59.0–70.0)	**0.** **034**
EXTEM CFT (s)	53.1 ± 19.6, 51.5 (47.0–55.0)	88.9 ± 5.7, 90.0 (86.0–93.0)	**<0.001**
EXTEM A10 (mm)	62.9 ± 8.3, 64.0 (59.0–67.0)	52.0 ± 4.2, 51.0 (49.0–54.0)	**<0.001**
EXTEM MCF (mm)	69.5 ± 8.0, 70.0 (63.0–75.0)	58.9 ± 3.5, 59.0 (57.0–61.0)	**<0.001**
EXTEM Alpha angle (°)	80.4 ± 6.5, 80.0 (77.0–83.5)	70.8 ± 2.7, 70.5 (69.0–73.0)	**<0.001**
EXTEM LI60 (%)	92.5 ± 2.5, 93.0 (91.0–94.0)	90.6 ± 4.2, 91.0 (88.0–93.5)	**0.022**
INTEM CT (s)	179.6 ± 10.2, 178.0 (175.0 −182.0)	185.1 ± 5.2, 186.0 (182.0–189.0)	**<0.001**
INTEM CFT (s)	64.8 ± 15.1, 64.0 (61.0–68.0)	72.7 ± 4.8, 73.0 (70.0–74.0)	**<0.001**
INTEM A10 (mm)	66.7 ± 8.9, 67.0 (62.5–71.0)	55.6 ± 4.1, 57.0 (51.0–58.0)	**<0.001**
INTEM MCF (mm)	70.9 ± 7.9, 72.0 (67.0–75.0)	59.0 ± 4.0, 59.0 (58.0–60.0)	**<0.001**
INTEM Alpha angle (°)	82.0 ± 5.8, 82.0 (80.0–84.0)	76.0 ± 4.1, 76.0 (74.0–78.0)	**<0.001**
INTEM LI60 (%)	93.6 ± 3.2, 94 (92.0–96.0)	88.0 ± 3.5, 88.0 (85.0–91.0)	**<0.001**

Data are presented as means ± SD, medians, and interquartile ranges (IQR). The nonparametric Wilcoxon rank sum test was used for the comparison between the 2 groups. Abbreviations: CT, clotting time; CFT, clot formation time; A10, clot amplitude at 10 min; MCF, maximum clot firmness; LI60, lysis index at 60 min.

**Table 4:** Results of multivariable linear regression analysis for ROTEM parameters as dependent variable with presence of bone tumor, age, gender, BMI, and smoking as independent variables.

Variables	Bone Tumor		
	Coefficient	95% CI	*p*-Value
EXTEM CT (s)	−3.62	−6.95 to −0.28	**0.034**
EXTEM CFT (s)	−34.71	−41.67 to −27.75	**<0.001**
EXTEM A10 (mm)	+10.71	+7.31 to +14.11	**<0.001**
EXTEM MCF (mm)	+10.46	+7.27 to +13.64	**<0.001**
EXTEM alpha angle (°)	+10.11	+7.64 to +12.57	**<0.001**
EXTEM LI60 (%)	+1.92	+0.28 to +3.56	**0.022**
INTEM CT (s)	−5.43	−9.67 to −1.19	**0.013**
INTEM CFT (s)	−6.50	−11.49 to −1.52	**0.011**
INTEM A10 (mm)	+11.32	+7.69 to +14.94	**<0.001**
INTEM MCF (mm)	+12.20	+8.92 to +15.47	**<0.001**
INTEM alpha angle (°)	+6.53	+3.88 to +9.18	**<0.001**
INTEM LI60 (%)	+5.42	+3.71 to +7.12	**<0.001**

Abbreviations: CI, confidence interval; CT, clotting time; CFT, clot formation time; A10, clot amplitude at 10 min; MCF, maximum clot firmness; LI60, lysis index at 60 min.

**Table 5:** EXTEM and INTEM parameters in patients with bone tumors before and after surgery.

Variables	Preoperatively (*n* = 50)	Postoperatively (*n* = 50)	*p*-Value
EXTEM CT (s)	61.2 ± 6.2, 61 (58.0–65.0)	56.3 ± 8.9, 56 (51.0–62.0)	**0.003**
EXTEM CFT (s)	53.1 ± 19.6, 51.5 (47.0–55.0)	48.9 ± 9.7, 47.0 (45.0–50.0)	**0.002**
EXTEM A10 (mm)	62.9 ± 8.3, 64.0 (59.0–67.0)	67.4 ± 8.0, 68.0 (62.0–75.0)	**0.007**
EXTEM MCF (mm)	69.5 ± 8.0, 70.0 (63.0–75.0)	74.0 ± 5.5, 75.0 (71.0–78.0)	**0.003**
EXTEM LI60 (%)	92.5 ± 2.5, 93.0 (91.0–94.0)	92.8 ± 2.3, 93.0 (91.0–95.0)	0.33
INTEM CT (s)	179.6 ± 10.2, 178 (175 −182)	175.8 ± 11.7, 175 (170.0–181.5)	**0.** **013**
INTEM CFT (s)	64.8 ± 15.9, 64.0 (61.0–68.0)	60.7 ± 12.1, 60.0 (54.5–67.0)	**0.018**
INTEM A10 (mm)	66.7 ± 8.9, 67.0 (62.5–71.0)	71.0 ± 6.7, 71.5 (67.0–76.0)	**0.009**
INTEM MCF (mm)	70.9 ± 7.9, 72 (67.0–75.0)	75.7 ± 6.6, 77 (73.0–79.0)	**0.005**
INTEM LI60 (%)	93.6 ± 3.2, 94 (92.0–96.0)	94.3 ± 3.0, 94.5 (93.0–97.0)	0.08

Data are presented as means ± SD, medians, and interquartile ranges (IQR). The nonparametric Wilcoxon signed-rank test was used for the comparison. Abbreviations: CT, clotting time; CFT, clot formation time; A10, clot amplitude at 10 min; MCF, maximum clot firmness; LI60, lysis index at 60 min.

**Table 6:** EXTEM and INTEM parameters in patients with metastatic and primary musculoskeletal tumors.

Variables	Metastastic (*n* = 12)	Primary (*n* = 18)	*p*-Value
EXTEM CT (s)	62.0 ± 3.1, 61.5 (59.5–65.0)	60.9 ± 7.1, 61.0 (56.0–65.0)	0.54
EXTEM CFT (s)	46.2 ± 11.1, 48.5 (36.0–54.5)	57.4 ± 19.5, 52.0 (48.0–58.0)	0.15
EXTEM A10 (mm)	72.3 ± 4.6, 73.5 (68.0–77.0)	59.2 ± 6.3, 62.0 (52.0–64.0)	**<0.001**
EXTEM MCF (mm)	79.1 ± 3.8, 79.5 (76.0–81.0)	65.8 ± 5.9, 68.0 (60.0–71.0)	**<0.001**
EXTEM LI60 (%)	92.9 ± 2.1, 92.0 (91.0–95.0)	92.4 ± 2.6, 93.0 (91.0–94.0)	0.81
INTEM CT (s)	176.6 ± 6.6, 177 (170 −183)	180.8 ± 11.1, 178 (176.0–182.0)	0.34
INTEM CFT (s)	63.0 ± 5.7, 62.0 (62.0–65.0)	67.6 ± 14.1, 64.0 (61.0–68.0)	0.30
INTEM A10 (mm)	70.7 ± 8.9, 72.0 (66.5–73.0)	65.2 ± 8.6, 67.0 (62.0–69.0)	**0.040**
INTEM MCF (mm)	79.2 ± 4.8, 80.0 (75.0–82.0)	67.8 ± 6.4, 71 (61.0–72.0)	**<0.001**
INTEM LI60 (%)	94.2 ± 3.0, 95 (93.0–97.0)	93.0 ± 3.1, 94.0 (92.0–95.0)	0.49

Data are presented as means ± SD, medians, and interquartile ranges (IQR). The nonparametric Wilcoxon signed-rank test was used for the comparison. Abbreviations: CT, clotting time; CFT, clot formation time; A10, clot amplitude at 10 min; MCF, maximum clot firmness; LI60, lysis index at 60 min.

## Data Availability

The data presented in this study are available on request from the corresponding author.

## Supplementary Materials

The following supporting information can be downloaded at: https://www.mdpi.com/article/10.3390/cancers14163930/s1, Table S1: The intra–assay coefficients of variation of ROTEM analysis for 15 patients; Table S2: Duplicate ROTEM measurements in 15 specimens.
