# Supplementary material for: The Hypercoagulable Profile of Patients with Bone Tumors: A Pilot Observational Study Using Rotational Thromboelastometry
Source: Cancers (Basel). 2022 Aug 15;14(16):3930. doi: 10.3390/cancers14163930 (PMC9406421; doi:10.3390/cancers14163930)
Supplement: Supplementary file 1 [file cancers-14-03930-s001.zip › cancers-1833431-supplementary.pdf]

Supplementary Table S1. The intra–assay coefficients of variation of ROTEM analysis for 15 patients.

Parameter	Intra–assay CV (%, 95% CI)
EXTEM CT	3.67 (0.58–5.25)
EXTEM CFT	2.47 (0.22–4.60)
EXTEM A10	2.45 (0.50–5.11)
EXTEM MCF	1.70 (0.50–4.35)
EXTEM alpha angle	1.52 (0.87–3.51)
EXTEM LI60	3.45 (0.50–6.54)
INTEM CT	2.65 (0.51–6.02)
INTEM CFT	4.52 (1.01–6.45)
INTEM A10	2.48 (0.45–4.67)
INTEM MCF	3.11 (0.50–7.75)
INTEM alpha angle	2.47 (0.25–4.99)
INTEM LI60	3.56 (1.24–6.88)

Abbreviations: CV, coefficient variation; CI, confidence interval; *Abbreviations:* CT, clotting time; CFT, clot formation time; A10, clot amplitude at 10 min; MCF, maximum clot firmness; LI60, lysis index at 60 min;

Supplementary Table S2. Duplicate ROTEM measurements in 15 specimens

Parameter	Specimens														
	1	2	3	4	5	6	7	8	9	10	11	12	13	14	15
EXTEM CT	61<br>62	70<br>71	64<br>65	81<br>82	71<br>73	54<br>54	67<br>66	48<br>49	57<br>55	81<br>80	66<br>66	50<br>51	45<br>47	66<br>65	62<br>63
EXTEM CFT	53<br>55	63<br>64	50<br>52	57<br>55	52<br>51	59<br>59	60<br>61	42<br>41	40<br>43	29<br>29	72<br>72	50<br>51	66<br>65	50<br>52	53<br>53
EXTEM A10	62<br>60	67<br>66	62<br>60	64<br>63	61<br>60	54<br>55	78<br>79	84<br>84	56<br>55	89<br>87	82<br>81	56<br>55	60<br>61	63<br>62	59<br>58
EXTEM MCF	69<br>70	80<br>81	59<br>59	45<br>46	68<br>70	89<br>88	39<br>40	55<br>55	78<br>77	79<br>79	67<br>66	65<br>64	50<br>51	54<br>55	66<br>67
EXTEM alpha angle	80<br>81	90<br>92	66<br>65	70<br>71	56<br>55	53<br>52	67<br>68	70<br>72	67<br>68	78<br>78	56<br>57	93<br>92	67<br>68	92<br>93	84<br>83
EXTEM LI60	92<br>94	93<br>91	96<br>96	97<br>98	90<br>89	90<br>92	93<br>93	91<br>92	94<br>95	98<br>98	79<br>81	87<br>88	89<br>88	91<br>92	93<br>94
INTEM CT	179<br>177	181<br>183	165<br>160	170<br>178	177<br>178	167<br>170	179<br>179	181<br>183	180<br>181	184<br>186	167<br>168	170<br>171	169<br>169	145<br>144	155<br>156
INTEM CFT	64<br>64	67<br>66	57<br>58	60<br>61	69<br>69	71<br>71	44<br>43	53<br>52	68<br>66	83<br>82	54<br>53	49<br>49	51<br>52	67<br>67	58<br>58
INTEM A10	68<br>65	70<br>72	64<br>63	65<br>66	48<br>49	72<br>73	89<br>89	64<br>64	56<br>57	60<br>61	63<br>64	51<br>53	52<br>63	69<br>67	62<br>63
INTEM MCF	70<br>71	73<br>73	69<br>71	67<br>67	68<br>68	79<br>79	91<br>92	71<br>72	68<br>69	62<br>63	70<br>71	58<br>58	57<br>58	74<br>75	69<br>70
INTEM alpha angle	82<br>82	84<br>86	80<br>81	92<br>94	95<br>90	67<br>68	79<br>78	81<br>82	72<br>73	68<br>68	89<br>90	93<br>95	78<br>79	84<br>84	88<br>88

INTEM LI60	92<br>94	93<br>94	90<br>89	97<br>99	91<br>94	92<br>93	94<br>94	87<br>86	96<br>95	90<br>87	92<br>94	91<br>92	96<br>98	99<br>99	91<br>92
Abbreviations: CT, clotting time; CFT, clot formation time; A10, clot amplitude at 10 min; MCF, maximum clot firmness; LI60, lysis index at 60 min;															
